# 3D Printable Graphene Composite

**DOI:** 10.1038/srep11181

**Published:** 2015-07-08

**Authors:** Xiaojun Wei, Dong Li, Wei Jiang, Zheming Gu, Xiaojuan Wang, Zengxing Zhang, Zhengzong Sun

**Affiliations:** 1 Department of Chemistry, Fudan University, Shanghai 200433, P. R. China; 2 Shanghai Key Laboratory of Special Artificial Microstructure Materials and Technology School of Physics Science and Engineering Tongji University, Shanghai 200092, P. R. China; 3 Department of Applied Chemistry Xi’an Jiaotong University, Shaanxi 710049, P. R. China; 4 Shanghai Key Laboratory for Engineering Materials Application and Evaluation, P. R. China; 5 Department of Chemistry and Shanghai Key Laboratory of Molecular Catalysis and Innovative Materials, Fudan University, Shanghai 200433, P. R. China

## Abstract

In human being’s history, both the Iron Age and Silicon Age thrived after a matured massive processing technology was developed. Graphene is the most recent superior material which could potentially initialize another new material Age. However, while being exploited to its full extent, conventional processing methods fail to provide a link to today’s personalization tide. New technology should be ushered in. Three-dimensional (3D) printing fills the missing linkage between graphene materials and the digital mainstream. Their alliance could generate additional stream to push the graphene revolution into a new phase. Here we demonstrate for the first time, a graphene composite, with a graphene loading up to 5.6 wt%, can be 3D printable into computer-designed models. The composite’s linear thermal coefficient is below 75 ppm·°C^−1^ from room temperature to its glass transition temperature (*T*_*g*_), which is crucial to build minute thermal stress during the printing process.

Although 3D printing technology has been around since the 1980 s, it hasn’t gained enough momentum to be commercialized until recently^1^,^2^. After the thermoplastic polymers, such as acrylonitrile-butadiene-styrene (ABS) and poly(lactic acid) (PLA), were introduced, 3D printing becomes more affordable through a fused deposition modelling (FDM) process. In a typical FDM process, thermoplastic polymer feedstock, usually a filament, is heated above its glass transition temperature (*T*_*g*_) and extruded through the 3D printer’s nozzle, whose diameter defines the printing resolution. Computer-designed 3D objects are constructed through successively stacking thermoplastic layers. The printing process not only is easy and rapid, but also can realize exotic topological structures which challenges the traditional casting and welding techniques.

Driven by new applications, the printable category keeps expanding into many frontier scientific and engineering fields^3^,^4^,^5^,^6^,^7^,^8^. But graphene, a single-atom-thick sp^2^ carbon crystal, has never been used as a 3D printing feedstock. Its two dimensional honeycomb structure endows it with many unique properties^9^,^10^,^11^, such as high Young’s modulus (~1 TPa)^12^, large specific surface area (maximum theoretical value ~ 2630 m^2^/g)^13^, remarkable thermal conductivity (~3000–5000 W/mK)^14^ and excellent electrical conductivity (~10^2^ S/m for reduced graphene oxide monolayers and 2 to 3 orders of magnitude higher for pristine graphene)^15^. To facilitate both, it becomes urgent to push graphene and related materials into the 3D printable category.

Starting with the graphene composites, graphene sheets have been successfully added into polymers to explore novel applications in mechanical reinforcement, flame retardant or electrical conductive composites^16^,^17^. In most previous studies, graphene fillers must be homogeneously dispersed in polymers for the optimal properties^11^. However, it’s still a major problem confronted by graphene composites that phase separation between graphene sheets and polymers usually occurs in synthetic process^18^,^19^. To minimize phase separation and obvious graphene aggregation, we used graphene oxide (GO) to substitute graphene as the starting filler material. GO contains oxygenated functional groups on its basal planes, which could improve graphene’s dispersion in polymer phases^9^,^20^,^21^,^22^,^23^, while these functional groups made GO electrical insulating due to the extensive presence of sp^3^ C-C bonds^24^,^25^. The electrical conductivity can be retrieved through restoration of sp^2^ bonds in graphitic domains. Chemical reduction of GO is a cost-effective strategy to prepare graphene sheets by utilization of reductant. Hydrazine has been commonly used to high yield production of reduced GO (rGO) owing to its ability to *in situ* reduce oxygenated functional groups. It was reported that rGO restores 80% sp^2^ in its structure, with the rest of sp^3^ bonds derived from residual oxygen (C:O atom ratio 12.5:1) after the reduction with hydrazine^25^. Herein, a series of graphene-based ABS and PLA composites were prepared through chemical reduction of GO by hydrazine and tested with a commercially available 3D printer.

Figure 1a-g demonstrate a series of images related to the process flow to prepare the 3D printable graphene-ABS (G-ABS) composite. Large graphite flakes were first oxidized into GO using an optimized method reported before^26^. Fully exfoliated GO sheets were dispersed in N-Methylpyrolidone (NMP) medium^27^ which is also compatible with the ABS polymer. The procedure of GO sheets dispersed in NMP is shown in the Supplementary Information. The two solutions (GO in NMP and ABS in NMP) were mixed using a homogenizer (Figure S1) at 15000 r.p.m. for 1 h, allowing a thorough mixing of GO sheets and the ABS. Figure 1d shows a homogeneous GO-ABS dispersion obtained through homogenizer mixing. Through chemical reduction of GO with hydrazine hydrate, black reduced G-ABS dispersion was *in-situ* formed without obvious phase separation. To fractionally precipitate G-ABS composites from NMP, the dispersion was added into water. The precipitated G-ABS composite is shown in Fig. 1f. The top layer is the G-ABS coagulations, while the bottom is the water phase. These composites were subsequently centrifuge-separated, washed and further dried in a vacuum oven. See Method Summary for composite preparation details. The high-speed homogenizer (Figure S1) is essential to yield homogeneous G-ABS composites. Phase separation between graphene and ABS polymers was observed without using the homogenizer during the synthetic process. A series of the composites with various graphene loadings were prepared using the above method. The reduced graphene oxide loading were 0.4, 0.8, 1.6, 2.3, 3.8, 5.6 and 7.4 wt%, respectively. A sample of 0.8 wt% graphene-PLA (G-PLA) composites was also prepared using the same strategy.

These graphene-polymer composites were further extruded into 1.75 mm diameter filaments to fit the commercialized 3D printer. As shown in Fig. 1h, a G-ABS filament was heated above its *T*_g_ in the heated build chamber (HBC). Subsequently, based on layer-by-layer manufacturing technique^1^,^2^, the graphene-based composite filament was extruded and deposited onto an 80 °C heated build platform (HBP) through a 0.4 mm diameter nozzle. Typical G-ABS composite with 3.8 wt% graphene loading were printed into 3D models and exhibited in Fig. 1i. All the G-ABS composites were tested with the 3D printing machine (Figure S2 and S3). Thermoplastic ABS was 3D printable, and its composites were also 3D printable after incorporation of graphene sheets. G-ABS composites with graphene loading below 5.6 wt% were smoothly extruded from the nozzle. Discontinuous extrusion was observed for the 7.4 wt% sample in 3D printing process, indicating material’s inhomogeneity. At a high graphene loading level (7.4 wt%), aggregated graphene sheets could clog the printer’s nozzle, resulting in the 3D printing failure. Up to date, the highest graphene loading in G-ABS composites used in our printer is up to 5.6 wt%. However, given a more powerful homogenizing or dispersing technique, the G-ABS’s 3D printing can certainly surpass this record. In addition to G-ABS composite, 0.8 wt% G-PLA filament was also prepared in this way and proved 3D printable. Its 3D printing feedstock and produced models were exhibited in Figure S4.

The graphene dispersion’s homogeneity directly affects the composite’s printing ability and other physical properties. With scanning electron microscopy (SEM), graphene individuals can be captured from the polymer matrix with different contrast and detailed morphologies. SEM images were taken from both the surface and the cross section of the composite to give a spatial perspective of the graphene dispersion inside composites (Figure S5 and S6). Graphene sheets featuring with lateral size around 3–5 μm were observed in Fig. 2a. Figure S5 shows G-ABS composites’ SEM images taken from the surface. When graphene loading is relatively low, bright graphene sheets sparsely scatter in the dark polymer context. As the graphene loading kept growing, a continuous and denser graphene network was formed in the polymer matrix. Composite with more graphene fillers showed higher electrical conductivity. However, aggregations was observed in the 7.4 wt% G-ABS sample (Figure S5c). Such aggregation is likely to account for 3D printing’s nozzle jam. The electrical conductivity (*σ*_*c*_) was investigated on hot pressed rectangular composite models using a four-probe method (Figure S7) and plotted against the graphene loadings (Fig. 2b) At 0.4 wt%, the electrical conductivity is 1.78 × 10^−7^ S·m^−1^. The value slowly climbed to 3.04 × 10^−7^ S·m^−1^ at 2.3 wt%. When graphene loading was up to 3.8 wt%, the conductivity drastically increased to 6.4 × 10^−5^ S·m^−1^. After it was 3D printed into 10 mm × 10 mm × 1 mm rectangular model (Figure S8), its measured conductivity decreased to 2.5 × 10^−7^ S·m^−1^. In FDM, the internal voids among adjacent stacked filaments (Figure S9) may account for the falloff in conductivity^3^,^28^. The highest graphene-loaded printable composite (5.6 wt%) bears a conductivity of 1.05 × 10^−3^ S·m^−1^. The G-ABS composite’s conductivity mechanism can be described with a bond percolation model^29^ (Figure S10). A sharp increase in composites’ electrical conductivity occurs once the graphene fillers form an infinite network^29^,^30^, which can be defined as the percolation threshold^31^ (*Φ*_c_). *Φ*_c_ in previous graphene-based polymer composite was reported to be around 0.1 vol%^30^,^32^,^33^. It could vary from different materials and preparation methods. In our G-ABS samples, the percolation threshold (the onset of the transition) is estimated to be ~0.9 vol%, which is ~2.0 in wt%. At least three of our G-ABS samples (2.3, 3.8 and 5.6 wt%) surpassed this threshold. This electrically conductive composite enables 3D printing to tackle with more complex engineering problems, such as building smart structures inside an insulating ABS models or printable electronics^29^,^34^.

Raman and ultraviolet-visible (UV-vis) analysis were used to further verify the presence of graphene in composites. A laser excitation with a wavelength of 785 nm was used to record Raman spectra from the samples (Fig. 2c). The representative spectrum of G-ABS (3.8 wt% graphene loading) shows a broad D peak at ~1330 cm^−1^ and a G peak at ~1600 cm^−1^, related to the defects and sp^2^ structures in graphene^35^ (Figure S12). A relative weak peak at *~*2240 cm^−1^ can be ascribed to the C≡N stretch in ABS^36^. These three peaks can be captured from the whole series Raman spectra of G-ABS samples in Figure S12. Detailed Raman peak positions and assignments of GO, rGO, pure ABS and G-ABS composites are listed in Table S1 and S2. In Fig. 2d, the UV-vis spectrum of GO displayed a characteristic absorption at ~231 nm, corresponding to the π-π* transition in GO. After chemical reduction with hydrazine, this peak red-shifted to ~265 nm, indicating the restoration of the conjugated structure in rGO^27^. The same signature peak at ~265 nm is also observed in a zoomed-in UV-vis spectrum for standard G-ABS samples (Figure S13).

Temperatures of nozzle, HBC and HBP are crucial parameters to smoothly print the composite filament without major structural flaws. To calibrate our 3D printing system for optimal performance, ABS and G-ABS samples’ thermal and mechanical properties were carefully assessed with standard techniques, such as differential scanning calorimetry (DSC), dynamic mechanical analysis (DMA), thermogravimetric analysis (TGA) and thermomechanical analysis (TMA). In FDM printing, printing materials need to be heated above *T*_*g*_ and then cool down from it to room temperature (RT). Therefore, before any 3D printing, *T*_*g*_ is the first and most vital parameter to be determined^37^. Fig. 3a is the representative DSC curves, from which *T*_*g*_ values can be calculated and listed in Table S3^32^. Pure ABS has a *T*_*g*_ value close to ~105.8 °C. This value slightly shifted to ~110 °C with incremental graphene loadings (Table S3). The reinforcing graphene network might lead to enhancement of *T*_*g*_^38^. Graphene additives restrict the segmental mobility of ABS’s chain segments near graphene sheets, which increases the melting temperature of the amorphous materials^32^,^39^. Same trend was observed in DMA-derived *T*_*g*_ values, with a slight elevation as the graphene loading increases (Table S3).

It was expected that polymer’s viscoelasticity would change after the incorporation of graphene sheets. The loss factor (tan δ), ratio of loss and storage modulus, is usually a characteristic indicator of how much energy dissipates in the polymer matrix^32^. Its peak maximum occurs at the transition regime from polymer’s glassy to rubbery state^32^,^40^ (Fig. 3b). It is known that large tan δ value reflects a viscous behavior whereas small tan δ indicates an elastic behavior^32^. The presence of graphene in ABS clearly lowered the maximum tan δ value, from ~2.3 for pure ABS to less than 0.5 for 7.4 wt% G-ABS. Graphene sheets highly influenced the molecular dynamics in the composites, giving rise to increasing the *T*_*g*_ and thermal stability of the composites as compared to the pure ABS polymers^32^. The storage modulus was measured at 1 Hz to analyze the influence of rGO reinforcement in ABS (Figure S14). The storage modulus results showed that G-ABS composites are higher than pure ABS polymer in the glass regime between 102 °C and 113 °C. The DMA results of G-ABS composites demonstrated a more elastic behavior as compared with pure ABS, suggesting an enhanced stiffness of the material.

The HBC needs to get hot enough to soften the composites^2^,^37^. But if the temperature is set too high, it could degrade the polymer ingredients^2^,^40^. In this case, thermal stability of the samples were further investigated by TGA to ensure the composite filaments were stable and merely softened rather than decomposed in 3D printer’s HBC. Under pyrolytic conditions in N_2_ atmosphere, the degradation of the samples occurred with a sharp weight loss around 400 to 480 °C (Fig. 3c and Figure S15), accompanied with possible evolved organic fragments (e.g. styrene, toluene, propenylbenzene etc.)^41^. The degradation onset temperature (T_onset_) is defined as the temperature when testing sample loses 5% of its total weight^16^. As summarized in Table S3, the lowest onset temperature of these composite samples is 339.17 °C. Since the HBC temperature was set at 230 °C, which was more than 100 °C below the T_onset_, the composite materials simply melt into viscous fluids rather than decomposing. The set temperature was also sufficiently higher than the G-ABS’s *T*_*g*_, ensuring the filaments smoothly printed through the 3D printer.

Any temperature change in thermoplastic is accompanied with thermal deformation, either expansion or contraction^42^. In 3D printing, material with large coefficient of thermal expansion (CTE) could build higher thermal stress and deform the printed structures during the cooling process. With TMA measurement in Fig. 3d, all samples including ABS and its graphene composites exhibited less than 1% expansion from RT to *T*_*g*_. CTE values were extracted in the same temperature range, according to CTE = (*dL*/*L*_*0*_)·(1/*dT*), where *dL*/*L*_*0*_is the dimensional deformation along the vertical axis and *dT* is the temperature change. All G-ABS samples’ CTE values were below 75 ppm·°C^−1^, which exhibited no significant variation from pure ABS’s 78.25 ppm·°C^−1^, suggesting suitable for the 3D printing. With *T*_*g*_ values ranging from 104 to 110 °C, by calibrating the HBP temperature at ~80 °C, 20 to 30 °C below *T*_*g*_ and maintaining the nozzle and HBC temperature at 130 °C and 230 °C, separately, we can efficiently avoid thermal deformation during the printing process (Figure S16).

In the past few years, both graphene and 3D printing have experienced a series breakthroughs in their own field. Our research presents one of the first attempts to directly print graphene related materials in 3D computer-designed fashions. These graphene-polymer composites were prepared using a solution based process, which can be easily scaled up to industry level. Upon further integrations, a whole spectrum of applications and functionalities based on these two leading technologies could be exploited.

## Methods summary

### Preparation of 3D printable graphene composite filament

The GO was prepared from a reported protocol^26^. In a typical synthesis of 3.8 wt% G/ABS composites, 30 ml GO-NMP (5 mg/ml) and 200 ml ABS-NMP (15 mg/ml) precursor were homogenized using a FJ300-S digital disperser with ø 28 mm dispersing element at 15000 r.p.m. Subsequently, chemical reduction of GO to graphene sheets was carried out with 0.5 ml 50% hydrazine hydrate at 95 °C for 1 h under homogenizing. Upon completion, the dispersion was slowly added to 400 ml DI water to precipitate the composites from NMP. The G-ABS composites were centrifuged at 4000 r.p.m. for 5 min, washed with 200 ml ethanol (2×) followed with 400 ml water (3×) and dried in a 120 °C oven for 24 h. The obtained composites were extruded from a single-screw extruder (LME, ATLAS, USA) into a 1.75 mm diameter filament at 210 °C. G-PLA filament was also prepared *via* the same process except ABS was replaced by PLA precursor.

### 3D Printing of graphene composite

A 3D printer (HOF1-X1, Nanjing Baoyan Automation Co., LTD, China) with a printing area of 140 mm x 180 mm x 135 mm was used to print our graphene related materials. The nozzle can move back and forth at a rate of ∼20 mm/s with a horizontal resolution of 200 μm or 400 μm (depending on the inner diameter of the nozzles) and vertical resolution of 200–400 μm to print out 3D models. The temperatures of HBC, HBP and nozzle were set at 230, 80 and 130 °C, respectively, for G-ABS 3D printing. They were changed to 190, 60 and 130 °C, respectively, for G-PLA 3D printing.

**Full Methods** and details of characterization for G-ABS and G-PLA composites are given in the Supplementary Information. A video showing the 3D printing process (3.8 wt% G-ABS) is also available in the Supplementary Information.

## Additional Information

**How to cite this article**: Wei, X. *et al.* 3D Printable Graphene Composite. *Sci. Rep.* **5**, 11181; doi: 10.1038/srep11181 (2015).

## Supplementary Material

Supplementary Information

Supplementary Video S1

## Figures and Tables

**Figure 1:**
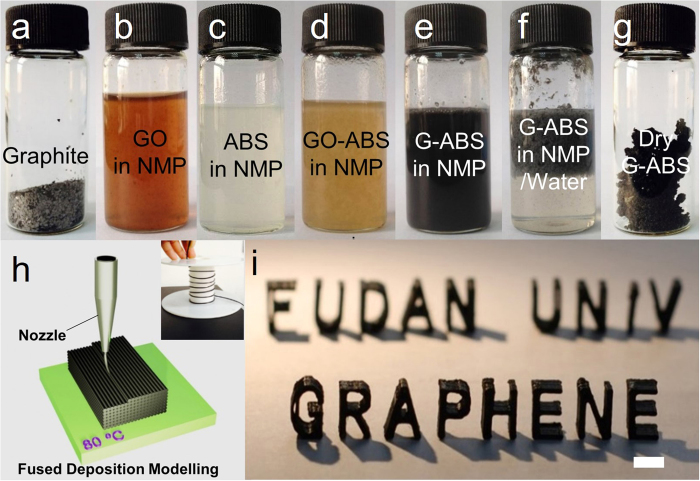
Composite preparation and 3D printing. **a**, Picture of graphite flakes. **b,c**, Dispersions of GO and ABS in NMP solvent. **d,e,** Pictures showing a homogeneous mixture of GO-ABS in NMP before and after chemical reduction by hydrazine hydrate at 95 °C for 1 h. Brownish GO-ABS turned into black G-ABS suspension during chemical reduction. **f**, G-ABS coagulations obtained after isolation (**e**) with water. **g**, G-ABS composite powder after washing and drying. **h**, Schematic illustration of fused deposition modelling 3D printing process. Inset is the graphene-based filament winding on a roller. The filament was deposited through a nozzle onto a heated building plated, whose temperature was set at 80 °C. **i**, A typical 3D printed model using 3.8 wt% G-ABS composite filament, scale bar: 1 cm.

**Figure 2:**
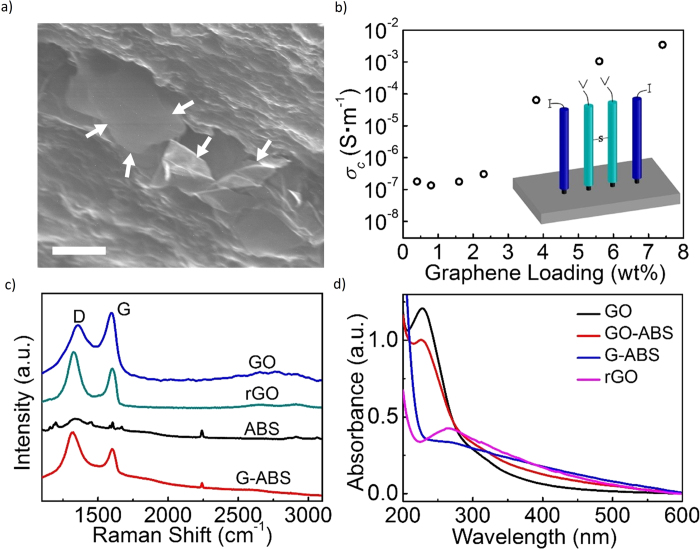
Spectroscopic and electrical analysis of graphene composites. **a**, A typical cross-section SEM image of 3.8 wt% G-ABS composite’s revealed partially incorporated dangling graphene sheets. **b**, Electrical conductivity (*σ*_*c*_) of G-ABS composites as a function of graphene loading. Inset is the four-probe schematic setup used in the *σ*_*c*_ measurement. **c**, Representive Raman spectra in prepared GO, rGO, ABS and G-ABS samples. **d,** UV-vis spectra of separated samples dispersed in aqueous solutions, including GO, GO-ABS, G-ABS, rGO.

**Figure 3:**
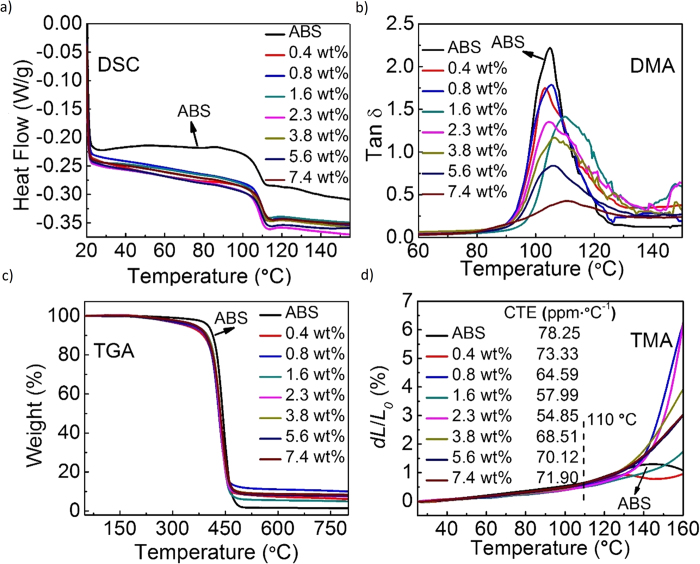
Thermal and mechanical analysis of ABS and G-ABS composites. **a**, Representative DSC curves of ABS and G-ABS composites. The T_g_ value slightly increased as the graphene loading in composites increased, calculated from the DSC curves. **b**, Loss factor (tan δ) derived from DMA. In all prepared samples, pure ABS claims the highest tan δ. by adding graphene sheets into ABS, ABS’s chain mobility was constrained with graphene’s stiff frameworks, which induces smaller tan δ values. **c,d**, TGA and TMA curves of ABS and G-ABS composites as a function of temperature. CTE values were calculated from the linear region (RT to 110 °C) in TMA.
