# Supplementary material for: 3D Printable Graphene Composite
Source: Sci Rep. 2015 Jul 8;5:11181. doi: 10.1038/srep11181 (PMC4495599; doi:10.1038/srep11181)
Supplement: Supplementary Information [file srep11181-s1.pdf]

3D Printable Graphene Composites-supplementary information

Xiaojun Wei[1], Dong Li[2], Wei Jiang[1,3], Zheming Gu[4], Xiaojuan Wang[2], Zengxing Zhang[2], Zhengzong Sun[1,5]*

[1] *Department of Chemistry, Fudan University, Shanghai 200433, P. R. China;*

[2] *Shanghai Key Laboratory of Special Artificial Microstructure Materials and Technology School of Physics Science and Engineering Tongji University, Shanghai 200092, P. R. China;*

[3] *Department of Applied Chemistry Xi'an Jiaotong University, Shaanxi 710049, P. R. China;*

[4] *Shanghai Key Laboratory for Engineering Materials Application and Evaluation, P. R. China.*

[5] *Department of Chemistry and Shanghai Key Laboratory of Molecular Catalysis and Innovative Materials, Fudan University, Shanghai 200433, P. R. China*

*Correspondence to: zhengzong_sun@fudan.edu.cn

1. Supplementary methods

1.1 Materials

1.2 Preparation of GO

1.3 Characterizations

2. Figures and tables

Figure S1 Optical images and scheme illustration of homogenizer.

Figure S2. Photographs of 3D printed models with different G-ABS composites.

Figure S3. Optical images of 3D printer and scheme illustrations.

Figure S4. Photographs of 3D printed models with 0.8 wt% G-PLA composites.

Figure S5. SEM images taken from the surfaces of G-ABS samples and an illustration of the graphene dispersions inside polymer.

Figure S6. Cross-sectional SEM images of G-ABS composites with different graphene loadings.

Figure S7. Arrangement of a four probe measurement on G-ABS samples.

Figure S8. A 3D printed cubic model by 3.8 wt% G/ABS composite.

Figure S9. Illustration of internal voids formation in 3D printing process.

Figure S10. Plot of the logarithm of dc conductivity (σ_c) vs the volume fraction (ϕ)

Figure S11. Zoomed in Raman spectra of 2D peaks.

Figure S12. Roman spectra of ABS and G-ABS samples.

Table S1. Characteristic Raman spectra bands for GO, graphene, ABS and G-ABS samples.

Table S2. Characteristic D, G and 2D bands for GO, rGO and G-ABS composites.

Table S3. T_g and T_{onset} obtained from DMA, DSC and TGA on ABS and G-ABS samples

Figure S13. UV-vis spectra of rGO (pink) and G-ABS composites (blue) in zoomed-in region.

Figure S14. Storage moduli (*E'*) as a function of temperature for G-ABS composites with different graphene loadings.

Figure S15. TGA curve of rGO.

Figure S16. Illustration of thermal warping in 3D printing.

3. Supplementary references

4. Video S1. A New 3D Printing Ink: Graphene Related Materials.

1. Supplementary methods

1.1 Materials

Graphite flakes (~10 mesh) were obtained from Alfa Aesar. H_2SO_4 (95-98%, AR) and HCl (36%, AR) were purchased from Suzhou Zhitang Chemicals Co., Ltd., China. H_3PO_4 (≥85%, AR) and H_2O_2 (30%, AR) were purchased from Jiangsu Tongsheng Chemical Reagent Co., Ltd., China. $KMnO_4$ was purchased from Shanghai Chemical Reagent Co., Ltd. Hydrazine hydrate (50%, AR), N-Methyl pyrrolidone (NMP) and ethanol (95%, AR) were all purchased from Sinopharm Chemical Reagent Co., Ltd., China. Acrylonitrile-butadiene-styrene (ABS, Mw≈120000-140000 g/mol) was

purchased from Zhenjiang Chimei Chemical Co., Ltd. China. Polylactic acid (PLA, Mw≈51000 g/mol) was purchased from Nature works chemicals Inc, USA.

1.2 Preparation of GO

Briefly, a mixture of H_2SO_4/H_3PO_4 (120:13.3 ml) was introduced to a mixture of 1 g graphite flakes and 6 g $KMnO_4$. The reaction was carried out at 50±2 ℃ and stirred for 12 h with speed of 180 r.p.m. Then the reaction was poured onto ~400 ml ice followed by addition of 1.5 ml H_2O_2 (30%). The mixture was left overnight, the supernatant was decanted and the bottom solids were washed with deionized water. The solid material was collected *via* centrifugation (4000 r.p.m., 30 min). The obtained material was further centrifuged in succession with 200 ml water, 200 ml 30% HCl, and 200 ml ethanol (2×) to decant away the impurities. GO/NMP solution was obtained by exchanging water in GO aqueous solution with NMP. GO aqueous solution was exchanged and centrifuged with NMP at 8000 rpm for 20 min per time. To insure NMP fully exchanged water, the procedure was repeated 10 times. GO was redispersed in N-Methyl-2-pyrrolidone (NMP), forming a 5 mg/ml GO-NMP solution. *Note we started from GO-NMP solution instead of dried rGO to get a homogenous GO-ABS mixture.*

To assess the rGO weight percentage, same amount of GO aqueous solution (made from 1 g graphite flake) was dried at 110 ^{o}C for 24 h, obtaining 1.5 g of product. Afterwards, 0.324 g dried GO was mixed in 200 NMP at 15000 r.p.m. with the homogenizer, followed by addition of 1 ml hydrazine. The reaction was performed at 95 ℃ for 2 h. Through centrifuging at 8000 r.p.m., washed ethanol (2×) and water(3×)

and dried in a 120 ℃ oven for 24 h, 0.257 g rGO (W_{rGO} = 0.79 W_{GO}) was obtained, where W_{GO} was weight of GO and W_{rGO} was weight of rGO. The GO loading were 0.5, 1.0, 2.0, 3.0, 4.8, 7.0 and 9.1 wt%, calculated from the starting material GO's weight. Correspondingly, The rGO weight percentage in the composite was calculated as follows: wt %=0.79W_{GO} /(0.79W_{GO}+ $W_{polymer}$), where $W_{polymer}$ was weight of polymer. The rGO loading were 0.4, 0.8, 1.6, 2.3, 3.8, 5.6 and 7.4 wt%, respectively.

1.3 Characterizations

SEM. SEM images were taken from freeze-fractured cross sections of the 4 mm×10 mm×0.5 mm G-ABS composites on Nova Nano SEM 450 at an acceleration voltage of 3 kV. Among these G-ABS composites, the 0.8 wt% and 2.3 wt% G-ABS composites were decorated with a thin layer of gold to minimize charging before imaging. **Electrical conductivity.** The polymer composites were hot pressed (XLB-D, Shanghai Rubber Machinery Works No.1 Co. Ltd, China) into 4 mm×10 mm×0.5 mm at 10 MPa with a temperature of 210 ℃. A 4 mm×10 mm×0.5 mm composite sample was cut into 2 mm wide and 10 mm long strips. The conductivity was measured on a 2mm×10 mm×0.5 mm strip. The d.c. resistance of the composites was recorded using a probe (ST 2558B-F01) and picoammeter (Keithley 4200), the electrical conductivity was then calculated, details of calculation were displayed in Figure S11. **Raman analysis.** Raman spectroscopy (Renishaw, 785 nm) was used to characterize the samples. For Roman analysis of reduced GO (rGO), rGO sample was prepared as follows. GO solution was chemically reduced to graphene by hydrazine. 3 ml GO water solution (5 mg/ml) was added in 50 ml water, the solution was heated to 95 ^{o}C

with stirring speed of 2000 r.p.m., followed by addition of 0.5 ml 50 % hydrazine hydrate. The reaction was kept for 1 h, then the product was collected via centrifugation (8000 r.p.m., 5 min). The black precipitates was washed with 100 ml water and 100 ml ethanol twice, respectively. Finally, the product was dried at 120 ℃ for 24 h. **UV-vis analysis.** UV-vis spectra (UV-3600, Shimazu, Japan) were obtained by a diluted aqueous solution of the samples. **TGA.** TGA (Pyris-1 TGA, Perkin Elmer) was performed from 50 to 800 ℃ at a heating rate of 10 ℃/min under nitrogen. **DSC.** T_g was determined using DSC (Q2000, TA Inst. Co.). Samples with 6 mm×6 mm×2 mm were performed in a two-step procedure: in first run**,** the composites were heated from 20 ℃ to 120 ℃ at a heating rate of 10 ℃/min, then cool down to room temperature at a cooling rate of 10 ℃/min; in second run, the samples were heated to 160 ℃ at a heating rate of 3 ℃/min. **TMA, DMA.** For TMA and DMA analysis, the polymer composites were hot pressed into 6 mm×6 mm×2 mm and 4 mm×10 mm×0.5 mm in a compression moulding machine at 10 MPa with a temperature of 210 ℃. TMA measurements were performed to record the deformation of the sample in the direction of the thickness using a penetration probe under a constant load of 0.01 N. CTE was determined by a thermomechanical analyzer (TMA/SDTA841e, Mettler Toledo, Inc., Switzerland). The samples with 6 mm×6 mm×2 mm were performed in a two-step procedure: in first run, the samples were heated to 120 ℃ with a heating rate of 10 ℃/min then cooled down to 25 ℃ with a cooling rate of 10 ℃/min to eliminate the influence of enthalpy relaxation; in second run, the samples were heated to 160 ℃ with a heating rate of 5 ℃/min. The CTE curves shown in this manuscript are traces

collected in second run. For DMA measurements, a dynamic mechanical analyzer (DMA/SDTA861e, Mettler Toledo, Inc., Switzerland) was used to characterize the mechanical property of the composite samples. The measurement of sample with 4 mm×10 mm×0.5 mm size was carried out in air from room temperature to 150 ℃ with a heating rate of 3 ℃/min at a constant frequency of 1 Hz.

2. Figures and tables

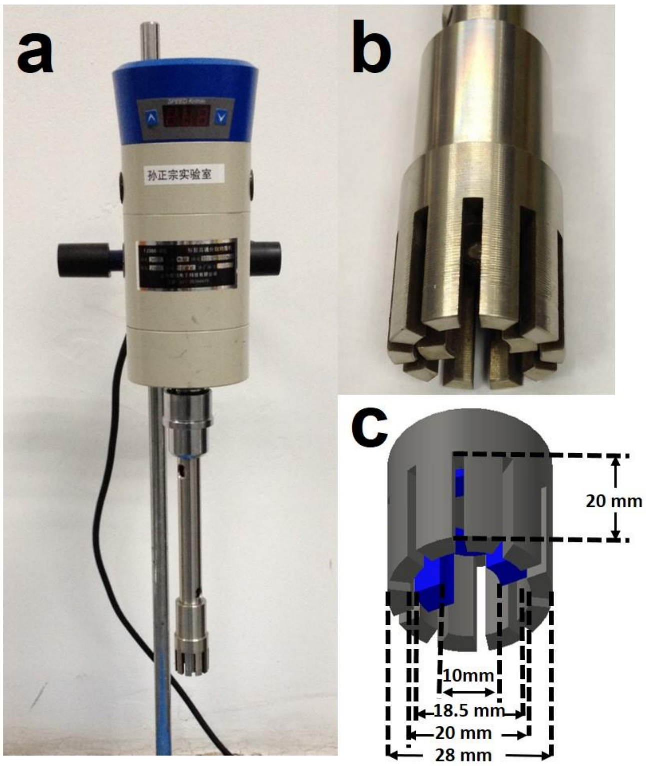

Figure S1. (a) A Picture of the homogenizer (Shanghai Specimen and Model factory). (b) A picture of the dispersing rotor and (c) its schematic illustration.

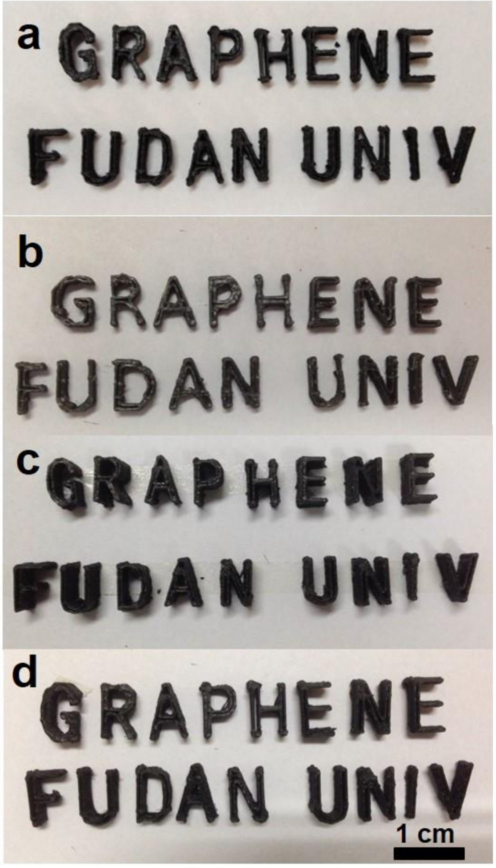

Figure S2. Photographs of 3D printed models using **(a)** 0.8 wt%, **(b)** 2.3 wt%, **(c)** 3.8 wt% and **(d)** 5.6 wt% G-ABS composite filaments.

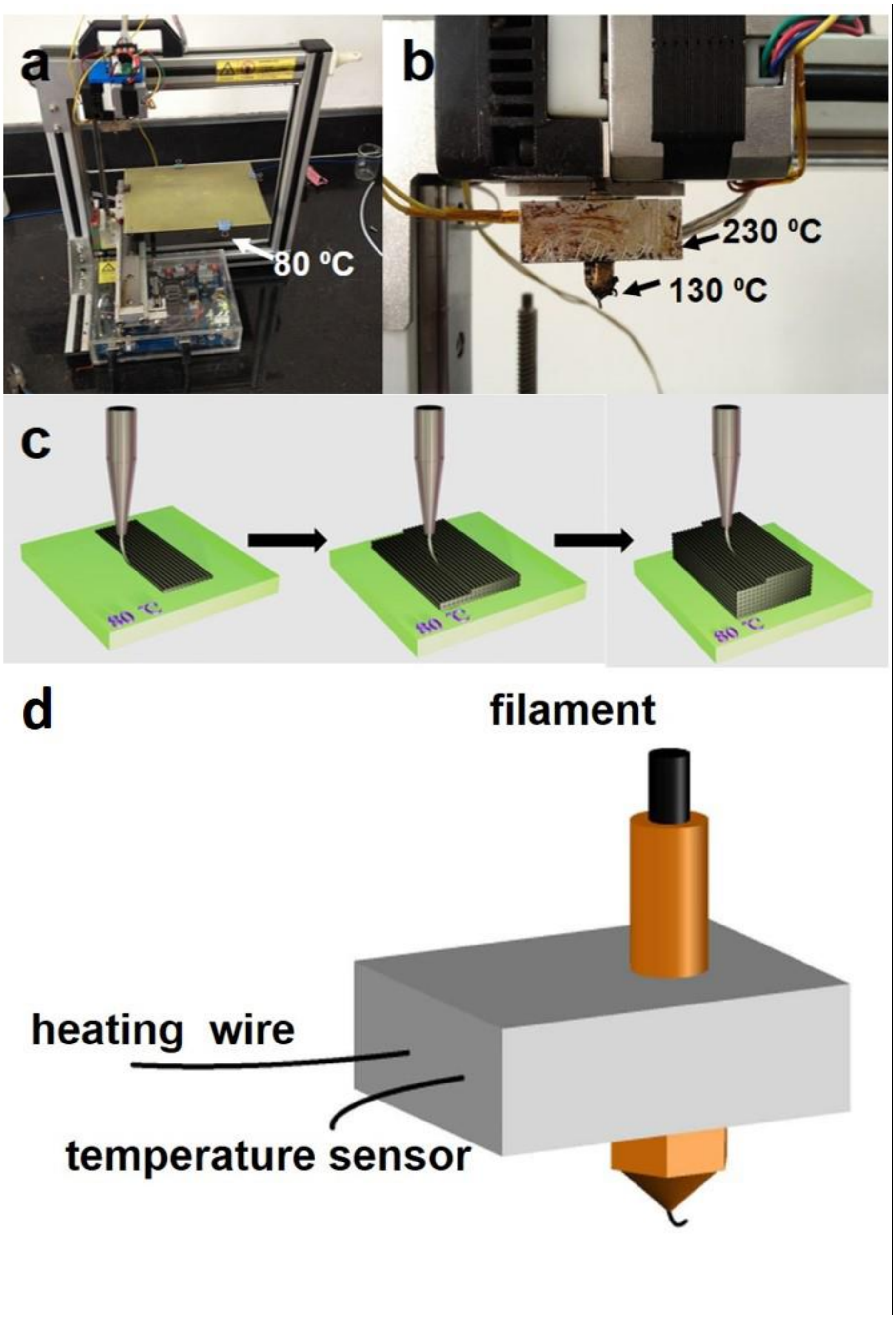

Figure S3. Optical images of the 3D printer **(a)** and nozzle **(b)**. 3D printing nozzle moved back and forth at rate ~20 mm/s. Scheme illustration of layer by layer manufacturing technique **(c)** and HBC **(d)**.

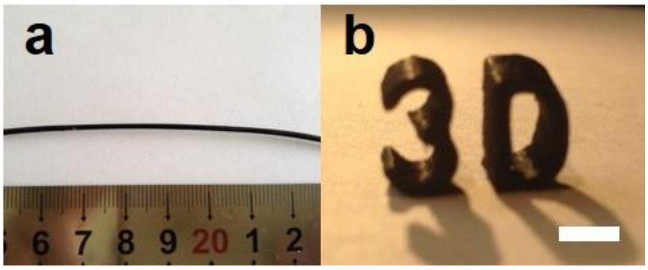

Figure S4. Photographs of **(a)** 0.8 wt% G-PLA filament and **(b)** corresponding 3D printed models.

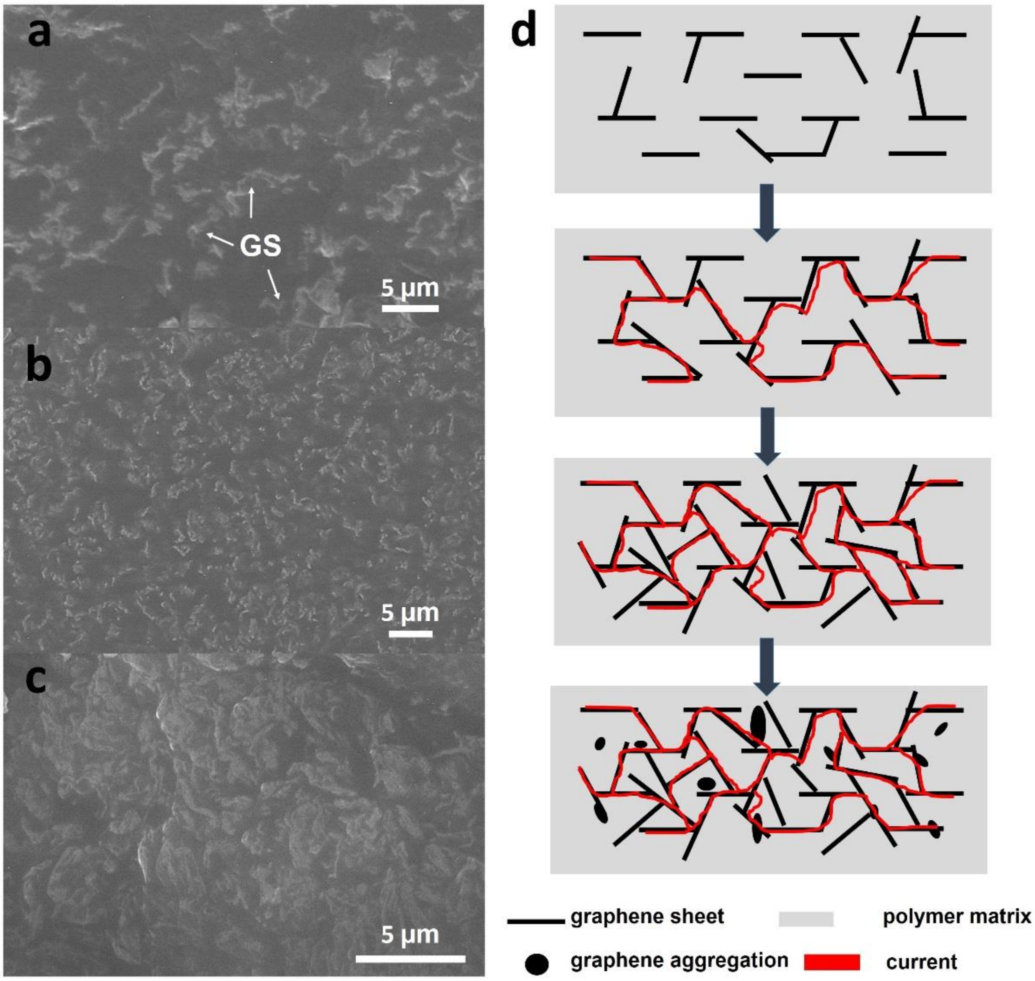

Figure S5. SEM images taken from the surfaces of **(a)** 2.3 wt%, **(b)** 3.8 wt% and **(c)** 7.4 wt% G-ABS composites, GS stands for graphene sheet. **(d)** Illustration of graphene dispersions in polymer. The composite material is insulating until graphene sheets form an infinate network of connected pathway to conduct electricity.

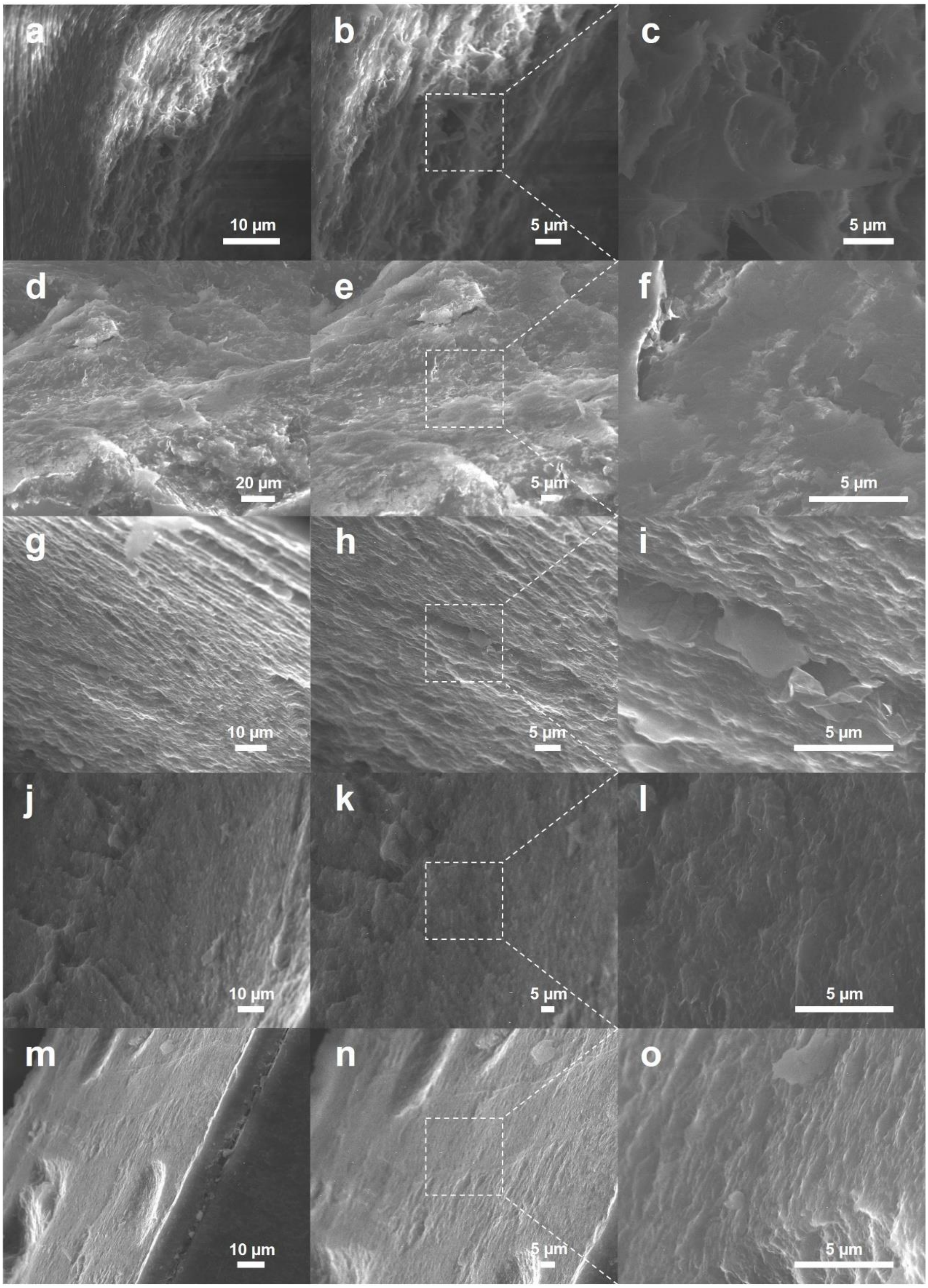

Figure S6. SEM images taken from cross-section of **(a, b, c)** 0.8 wt%, **(d, e, f)** 2.3 wt%, **(g, h, i)** 3.8 wt%, **(j, k, l)** 5.6 wt% and **(m, n, o)** 7.4 wt% G-ABS composites.

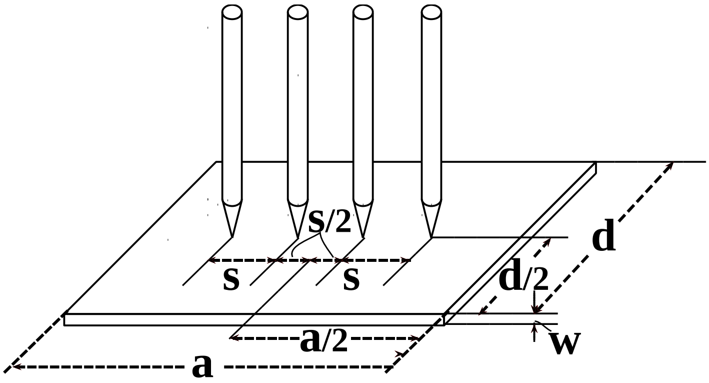

Figure S7. Arrangement of four probe measurement on G-ABS samples.

The electrical conductivity of the composites was calculated by a reported method for resistivity measurement.[1] The related equations are displayed below:

$$\rho = C \cdot \frac{V}{I} \cdot G\left(\frac{w}{s}\right) \cdot D\left(\frac{d}{s}\right)$$

$$C = 2\pi s$$

$$G\left(\frac{w}{s}\right) = 0.18 \text{ , } D\left(\frac{d}{s}\right) = 0.2205$$

$$\sigma = \frac{1}{\rho}$$

where

I ----current

V----voltage

ρ----resistivity

$G\left(\frac{w}{s}\right)$, $D\left(\frac{d}{s}\right)$ are correction factors[2] determined by: sheet thick (w), probe distance (s), length (d), and width (a) of sheet sample. Here s = 2 mm, a = 10 mm, and d = 2 mm, and w = 0.5 mm

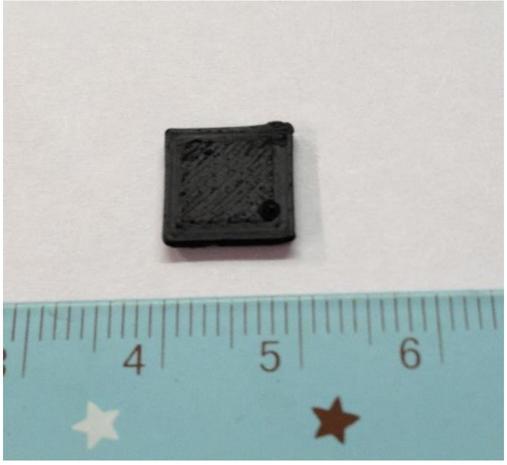

Figure S8. A 3D printed rectangular model by 3.8 wt% G/ABS composite.

According to the formula, $\rho = C \cdot \frac{V}{I} \cdot G\left(\frac{w}{s}\right) \cdot D\left(\frac{d}{s}\right)$

$$C = 2\pi s$$

$$G\left(\frac{w}{s}\right) = 0.429,\ D\left(\frac{d}{s}\right) = 0.2205$$

$$\sigma = \frac{1}{\rho}$$

where

I ----current

V----voltage

ρ----resistivity

$G\left(\frac{w}{s}\right)$, $D\left(\frac{d}{s}\right)$ are correction factors[2] determined by: sheet thick (w), probe distance (s), length (d), and width (a) of sheet sample. Here s = 2 mm, a = 10 mm, and d = 10 mm, and w = 1.2 mm. The conductivity reduced from 6.4×10^{-5} S/m to 2.5×10^{-7} S/m, ascribing to the formation of vacancies in 3D printing.

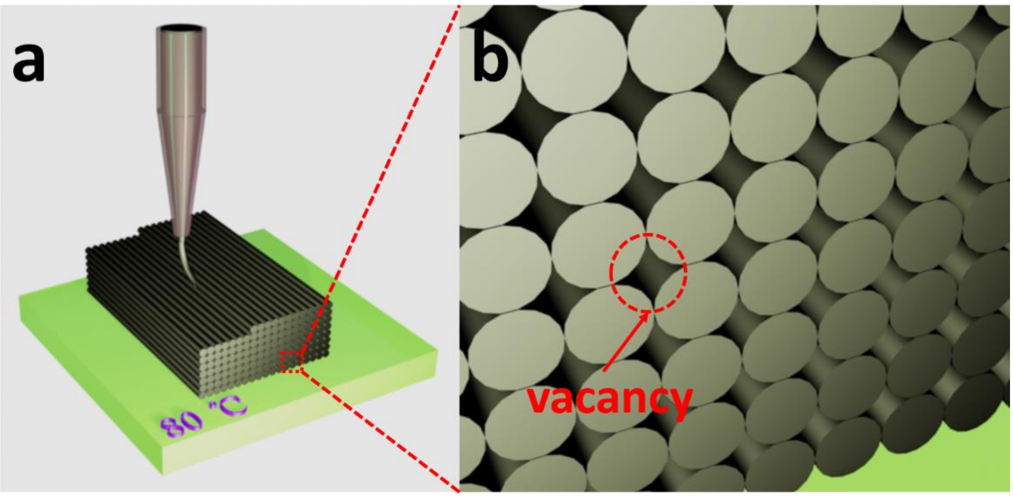

Figure S9. Illustration of internal voids formation in 3D printing process.

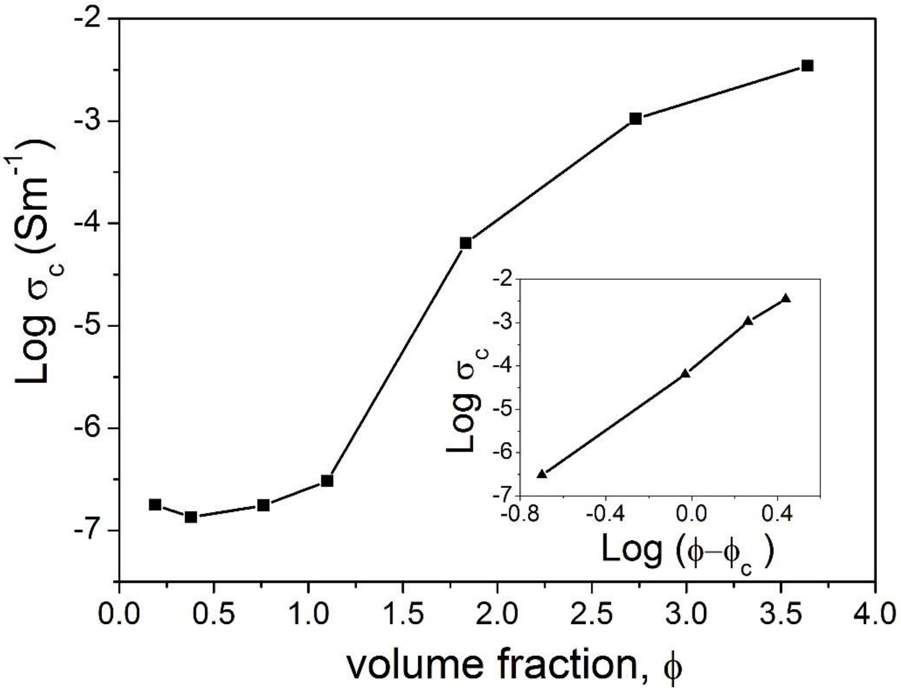

Figure S10. Plot of the logarithm of dc conductivity (σ_c) vs the volume fraction (ϕ) of graphene for the G-ABS composite system. The plot shows an open S curve or "sigmoid" curve[3]. According to the percolation theory, the conductivity (σ_c) of G-ABS composites is treated with the law: $\sigma_c = \sigma_f\,[(\phi - \phi_c)/(1 - \phi_c)]^t$ [30,31] when above the percolation threshold, where σ_f is the conductivity of graphene, ϕ is the graphene volume fraction, ϕ_c is the percolation threshold (chosen at the onset of the transition[30,35], ~ 0.9 vol%), and t is the "universal critical exponent". Inset is the log-log plot of conductivity against (ϕ- ϕ_c) of graphene in ABS system.

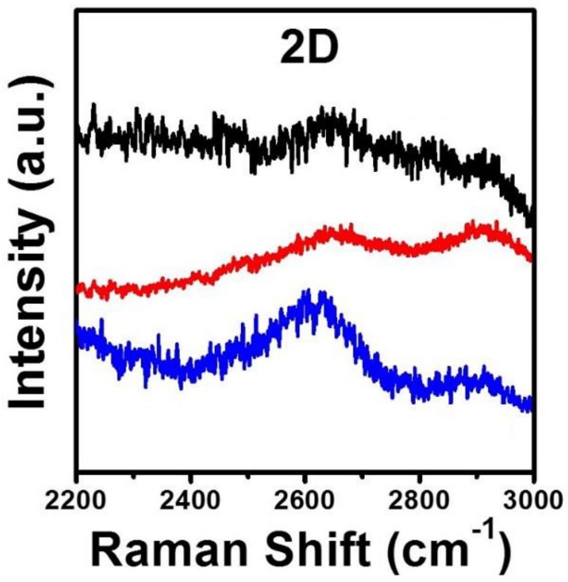

Figure S11. Zoomed in Raman spectra of 2D peaks. GO (black), rGO (red), and G-ABS composite (blue).

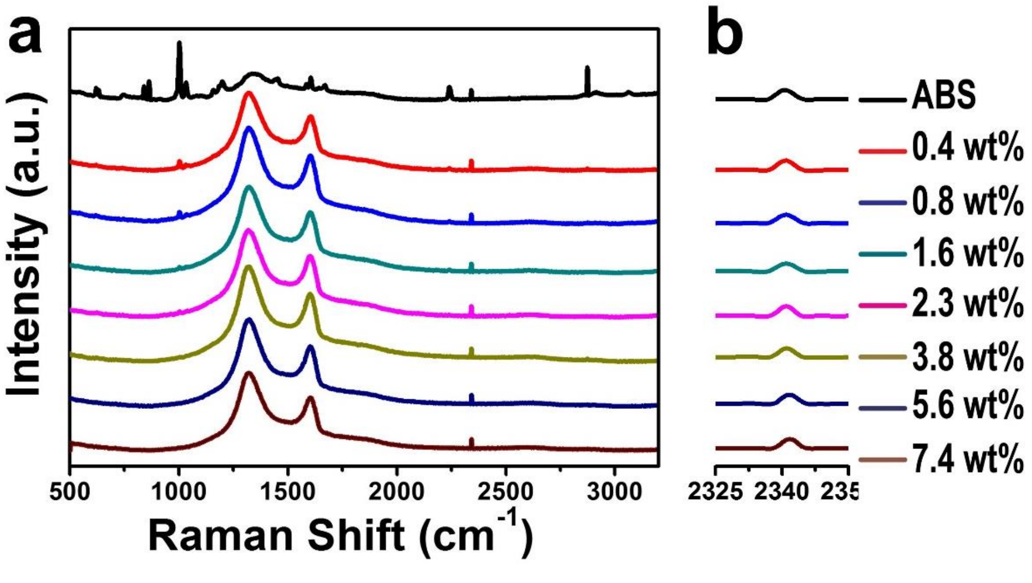

Figure S12. (a) Raman spectra of ABS and G-ABS composites. **(b)** Zoomed-in spectra around 2240 cm^{-1} region.

Table S1. Characteristic Raman spectra bands for GO, rGO, ABS and G-ABS composites.

Sample	Raman Shift (cm^{-1})	Assignment
GO	1357	D peak
	1596	G peak
rGO	1324	D peak
	1602	G peak
ABS[7]	2236	C≡N stretch (acrylonitrile)
G-ABS	~1320	D peak
	~1600	G peak
	~2240	C≡N stretch (acrylonitrile)

Table S2. Characteristic D, G and 2D bands for GO, rGO and G-ABS composites.

Sample	D peak (cm^{-1})	FWHM of D (cm^{-1})	G peak (cm^{-1})	FWHM of G (cm^{-1})	2D peak (cm^{-1})	FWHM of 2D (cm^{-1})	I_D/I_G
GO	1357	175	1596	113	2642	--	0.81
rGO	1324	106	1602	69	2651	115	1.40
G-ABS	1320	116	1600	59	2618	138	1.43

D peak is related to the breathing modes of sp2 atoms in hexagon rings activated by the defects such as vacancies or grain boundaries, while G peak is due to the bond stretching of all sp2 pairs in both rings and chains[4]. The ratio of D and G peak intensity, denoted as I_D/I_G, combined with their G peak's FWHM, are used to evaluate the structural disorders/perturbations[4-6]. As reported, the FWHM of G peak always increase with disorder, while I_D/I_G reaches to its maximum as the distance between defects, L_D, gets close to ~3 nm[5]. In our case as shown in Table S2, the I_D/I_G ratio of rGO and G-ABS were 1.40 and 1.43, respectively, higher than that of GO (I_D/I_G =0.81). Their FWHMs of G peak are 69 and 59 cm^{-1}, which are much smaller than GO's 113 cm^{-1}, indicating an increase of L_D upon reduction of GO by hydrazine[5]. Besides D and G peaks, 2D peak is the second order of the D peak which does not require any defect to be activated[5]. Its FWHM and dispersion are also important

indicators for graphene structure's disorders. In Figure S11, there was only a broad feature for GO from ~2300 to ~3000 cm^{-1}, where the 2D, D+D' and 2D' peaks were convoluted ambiguously, suggesting a highly disordered structure[5]. After the hydrazine reduction, both rGO and G-ABS showed a more distinctive 2D peaks with FWHMs around 115 and 138 cm^{-1}, respectively, suggesting the restoration of graphene structures[5].

Table S3. T_g and T_{onset} obtained from DMA, DSC and TGA on ABS and G-ABS composites

Sample	T_g (℃)		T_{onset}(℃)[c]	Resedue (wt%)[d]
	T_g–DSC[a]	T_g–DMA[b]		
ABS	106.81	104.89	398.76	1.4
rGO	-	-	-	72.1
0.4 wt% G-ABS	106.93	103.19	339.17	6.1
0.8 wt% G-ABS	108.51	105.34	349.74	10.0
1.6 wt% G-ABS	107.08	110.07	363.79	4.9
2.3 wt% G-ABS	107.72	104.55	357.05	7.4
3.8 wt% G-ABS	108.13	106.45	362.56	8.3
5.6 wt% G-ABS	108.46	106.36	359.2	8.4
7.4 wt% G-ABS	109.22	110.78	365.62	7.8

[a] T_g data is derived from DSC. [b] T_g data is measured by DMA. [c] The onset temperature of thermal degradation (T_{onset}) is designated as the temperature with 3.8 wt% weight loss. [d] TGA residue after being heated to 800 ˚C

As displayed in Table S2, discrepancy is discovered between the T_g values obtained from DMA analysis and DSC measurements, attributing to the fact that DMA is

highly dependent on the frequency of strain oscillation whereas DSC measures a heat quantity in a temperature change between the target sample and the reference sample, and variations are inevitable. Although the ABS and its composites T_g results obtained from these two means are not exactly identical, both show a tendency that the T_g value slightly increased as the graphene loading in composites increased.

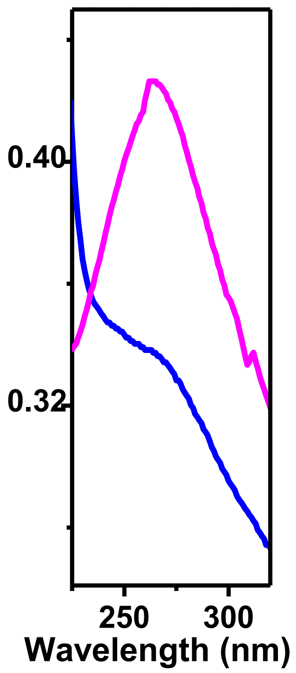

Figure S13. UV-vis spectra of rGO (pink) and G-ABS composites (blue) in zoomed-in region from Figure 2d.

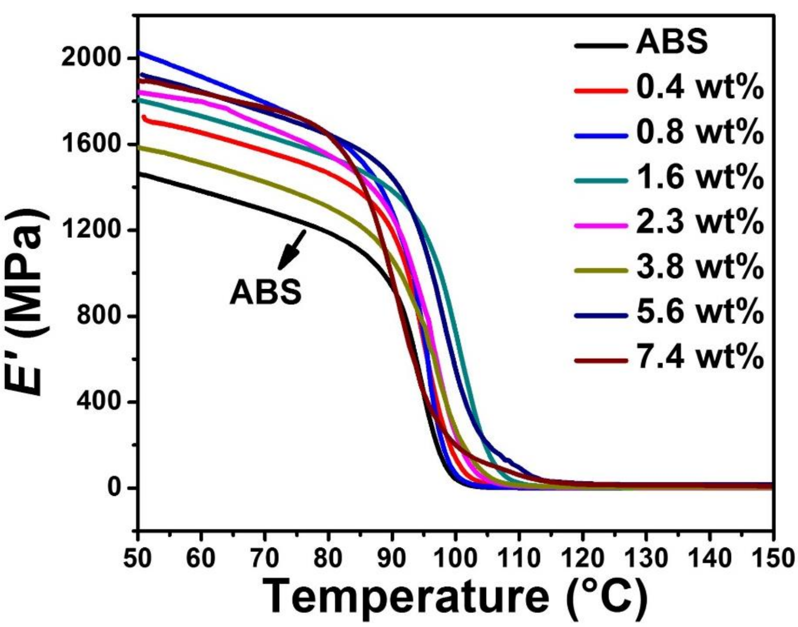

Figure S14. Storage moduli (*E'*) as a function of temperature for G-ABS composites with different graphene loadings.

DMA was performed at various temperature to determine the storage moduli (*E'*). It is observed that *E'* of G-ABS composites are higher than pure ABS polymer in the glass regime between 102 ℃ and 113 ℃. Also the *E'* values increase with an increasing graphene content in polymer composites although the increase is not in proportion to the amount of graphene fillers.

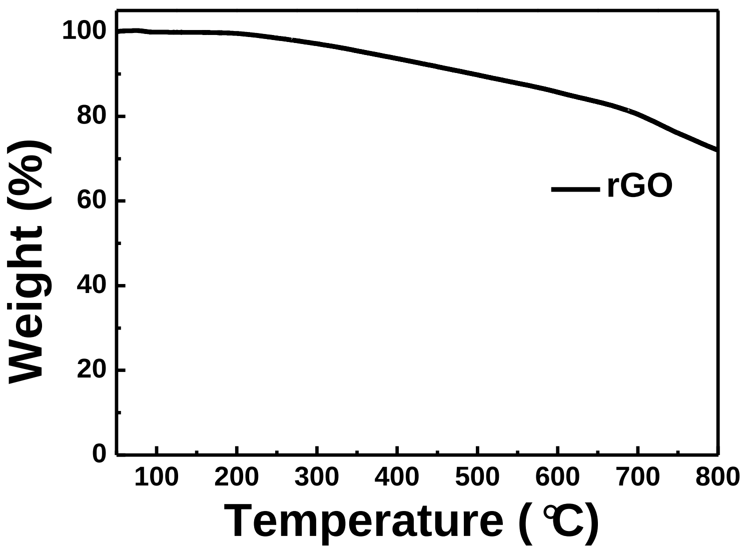

Figure S15. TGA curve of rGO.

The pure ABS had a loss weight of 98.4% after being heated to 800 ˚C under a nitrogen flow. For ABS and G-ABS samples, a mass weight loss occurred at 400 ˚C to 480 ˚C. The mass residue ranged from 6.1 wt% to 10.0 wt% for G-ABS samples after being heated to 800 ˚C (Table S3), ascribing to the removal of ABS in the composite. For rGO sample, the total weight loss was 28.9 wt% at 800 ˚C. This major loss could be attributed to the presence of functional groups on the rGO.

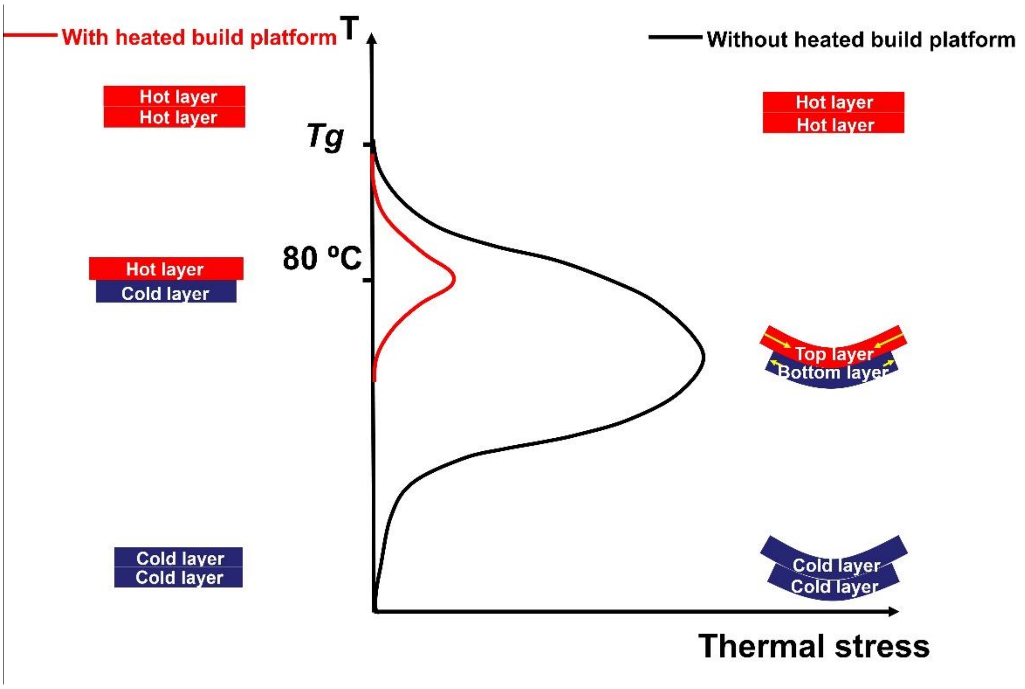

Figure S16. Illustration of thermal warping occurred in 3D printing.

During layer by layer process in 3D printing, a layer is ejected from hot chamber, it soon cools and shrinks. Subsequently, another hot layer is paved up and adhered onto the layer which is relatively cold, a steeper temperature gradient between these two layers leads to contraction of hot layer and compression of cold layer until these two layers reach thermal equilibrium, as illustrated in Figure S16. Therefore, thermal stresses are generated in this solidification process, inducing thermal warping in 3D printing. However, thermal stress can be suppressed by utilization of HBP. We set the HBP temperature at 80 ℃, approaching T_g of ABS at 106.81 ℃. In this case, this is an optimal temperature that ABS layer can be adhered on stiff plate without occurring thermal warping. In assistance with HBP (80 ℃), the stresses in plate itself compensate for the thermal stresses inside the ABS layer during 3D printing process.

Thermal stresses are vital to the quality of 3D printed prototypes because huge thermal stresses contribute to thermal warping. It is known that thermal stress (σ_t) is

proportional to CTE (α), temperature change below T_g (ΔT), and Young's modulus (E). The relationship is displayed following: $\sigma_t=\alpha\cdot E\cdot\Delta T$ [8]. Thus, to minimize the thermal contraction, it is feasible and practical to acquire relatively smaller constants of α and ΔT. The dimensional change from TMA measurements shows little variation at a temperature interval from room temperature to T_g. DMA and DSC results show that T_g values of all G-ABS composites are close to the pure ABS, so ΔT (here $\Delta T=T_g$-80) is regarded as a constant.

Supplementary references

1. Smits, F.M. Measurement of Sheet Resistivities with the Four-point Probe. *The bell system technical journal*. **20**, 711 (1958).
2. Topsoe, H. Geometric Factors in Four Point Resistivity Measurement. http://www.four-point-probes.com/haldor.html (1968).
3. Chiteme, C., McLachlan, D. S. AC and DC Conductivity, Magnetoresistance, and Scaling in Cellular Percolation Systems, *Phys. Rev. B.* **67**, 024206 (2003)
4. Ferrari, A. C. *et al.* Raman Spectrum of Graphene and Graphene Layers. *Phys. Rev. Lett.* **97**, 187401 (2006).
5. Cancado, L. G. *et al.* Quantifying Defects in Graphene via Raman Spectroscopy at Different Excitation Energies. *Nano Lett.* **11**, 3190–3196 (2011).
6. Ferrari A. C. Raman Spectroscopy of Graphene and Graphite: Disorder, Electron-Phonon Coupling, Doping and Nonadiabatic Effects. *Solid State Commun.* **143**, 47–57 (2007).

7. Stevanovic, D.; Lowe, A.; Kalyanasundaram, S.; Jar, P-Y.B.; Otieno-Alego, V. Chemical and Mechanical Properties of Vinyl-ester/ABS Blend. *Polymer* **43**, 4503–4514 (2002).
8. Lampman, S. Characterization and Failure Analysis of Plastics (ASM International, Ohio, 2003).

Video S1 shows the 3D printing process in printing 3.8 wt% G-ABS composite filament.
